# Reply to Post-traumatic Stress Disorder: A Major Source of Concern Comment on "Epidemiology of Post-traumatic Stress Disorder in Iranian Population From 2019 to 2024: A Systematic Review and Meta-analysis"

**DOI:** 10.34172/aim.33531

**Published:** 2025-02-01

**Authors:** Asad Imani, Shahram Molavynejad, Mojgan Khademi, Mohammad Adineh, Elham Shafiei, Mohsen Savaie

**Affiliations:** ^1^Student Research Committee, School of Nursing and Midwifery, Ahvaz Jundishapur University of Medical Sciences, Ahvaz, Iran; ^2^Nursing Care Research Center in Chronic Diseases, School of Nursing and Midwifery, Ahvaz Jundishapur University of Medical Sciences, Ahvaz, Iran; ^3^Community-Oriented Nursing Midwifery Research Center, Nursing and Midwifery School, Shahrekord University of Medical Sciences, Shahrekord, Iran; ^4^Social Determinants of Health Research Center, School of Nursing and Midwifery, Lorestan University of Medical Sciences, Khorramabad, Iran; ^5^Psychosocial Injuries Research Center, Ilam University of Medical Sciences, Ilam, Iran; ^6^Pain Research Center, Ahvaz Jundishapur University of Medical Sciences, Ahvaz, Iran

 We appreciate Santos et al^1^ for their meticulous attention to our published article.^2^ Iran is a country that is exposed to natural disasters^3^ and has had an 8-year war with Iraq; so, paying attention to post-traumatic stress disorder (PTSD) is always one of the concerns of researchers in this country. In this regard, PTSD has always been emphasized by Iranian research institutes. Since systematic review and meta-analysis articles are the basis for conducting other studies, including descriptive, analytical and clinical trial studies, we expect that our published article will attract the attention of researchers in future studies.

 In your letter to the editor, you mentioned several studies related to PTSD in Iran, all of which were conducted during 2024. You have shown the importance of the issue in Iranian research. We are pleased to published such studies that confirm the importance of our published article.
